# Evaluation of the efficacy and safety of icatibant and C1 esterase/kallikrein inhibitor in severe COVID-19: study protocol for a three-armed randomized controlled trial

**DOI:** 10.1186/s13063-021-05027-9

**Published:** 2021-01-20

**Authors:** Eli Mansour, Flávia F. Bueno, José C. de Lima-Júnior, Andre Palma, Milena Monfort-Pires, Bruna Bombassaro, Eliana P. Araujo, Ana Flavia Bernardes, Raisa G. Ulaf, Thyago A. Nunes, Luciana C. Ribeiro, Antônio Luís E. Falcão, Thiago Martins Santos, Plinio Trabasso, Rachel P. Dertkigil, Sergio S. Dertkigil, Rafael P. Maia, Tatiana Benaglia, Maria Luiza Moretti, Licio A. Velloso

**Affiliations:** 1 Department of Internal Medicine, School of Medical Sciences, University of Campinas, Campinas, São Paulo Brazil; 2 Obesity and Comorbidities Research Center, University of Campinas, Campinas, São Paulo Brazil; 3 School of Nursing, University of Campinas, Campinas, São Paulo Brazil; 4 Department of Surgery, School of Medical Sciences, University of Campinas, Campinas, São Paulo Brazil; 5 Department of Radiology, School of Medical Sciences, University of Campinas, Campinas, São Paulo Brazil; 6 Department of Statistics, Institute of Mathematics, Statistics, and Scientific Computation, University of Campinas, Campinas, São Paulo Brazil

**Keywords:** Pneumonia, ACE2, Bradykinin, Icatibant, C1 esterase inhibitor

## Abstract

**Background:**

SARS-CoV-2, the virus that causes COVID-19, enters the cells through a mechanism dependent on its binding to angiotensin-converting enzyme 2 (ACE2), a protein highly expressed in the lungs. The putative viral-induced inhibition of ACE2 could result in the defective degradation of bradykinin, a potent inflammatory substance. We hypothesize that increased bradykinin in the lungs is an important mechanism driving the development of pneumonia and respiratory failure in COVID-19.

**Methods:**

This is a phase II, single-center, three-armed parallel-group, open-label, active control superiority randomized clinical trial. One hundred eighty eligible patients will be randomly assigned in a 1:1:1 ratio to receive either the inhibitor of C1e/kallikrein 20 U/kg intravenously on day 1 and day 4 plus standard care; or icatibant 30 mg subcutaneously, three doses/day for 4 days plus standard care; or standard care alone, as recommended in the clinical trials published to date, which includes supplemental oxygen, non-invasive and invasive ventilation, antibiotic agents, anti-inflammatory agents, prophylactic antithrombotic therapy, vasopressor support, and renal replacement therapy.

**Discussion:**

Accumulation of bradykinin in the lungs is a common side effect of ACE inhibitors leading to cough. In animal models, the inactivation of ACE2 leads to severe acute pneumonitis in response to lipopolysaccharide (LPS), and the inhibition of bradykinin almost completely restores the lung structure. We believe that inhibition of bradykinin in severe COVID-19 patients could reduce the lung inflammatory response, impacting positively on the severity of disease and mortality rates.

**Trial registration:**

Brazilian Clinical Trials Registry Universal Trial Number (UTN) U1111-1250-1843. Registered on May/5/2020.

## Administrative information

**Table:** 

Title {1}	Evaluation of the efficacy and safety of icatibant and C1 esterase/kallikrein inhibitor in hospitalized patients with pneumonia caused by Covid-19 virus infection: a study protocol for a phase II, open-label, three-armed, randomized, single-center, active control superiority trial.
Trial registration {2a and 2b}	Brazilian Clinical Trials Registry (ReBec): RBR-5s2mqg Universal Trial Number (UTN): U1111-1250-1843
Protocol version [1]	Version 2.0 Nov. 18, 2020
Funding {4}	Funded by: Emergency project - Rapid Implementation Supplements Against COVID-19 of the Sao Paulo Research Foundation (FAPESP 2020/04522-5). Research, Inovation and Dissemination Center – Obesity and Comorbidities Research Center of the Sao Paulo Research Foundation (FAPESP 2013/07607-8).
Author details {5a}	Obesity and Comorbidities Research Center, University of Campinas, Campinas, São Paulo, Brazil. Department of Internal Medicine, School of Medical Sciences, University of Campinas, Campinas, São Paulo, Brazil. Department of Surgery, School of Medical Sciences, University of Campinas, Campinas, São Paulo, Brazil. Department of Radiology, School of Medical Sciences, University of Campinas, Campinas, São Paulo, Brazil. Clinical Hospital, University of Campinas, Campinas, São Paulo, Brazil. School of Nursing, University of Campinas, Campinas, São Paulo, Brazil.
Name and contact information for the trial sponsor {5b}	Clinics Hospital, University of Campinas, Campinas, São Paulo, Brazil, R. Vital Brasil, 251 - Cidade Universitária, Campinas - SP, 13083-888
Role of sponsor {5c}	The study sponsor and funders have no role in study design; collection, management, analysis, and interpretation of data; writing of the report; and the decision to submit the report for publication.

## Introduction

### Background and rationale {6a}

The acute respiratory illness caused by SARS-CoV-2 infection has been confirmed in + 3.3 million patients, leading to the deaths of more than 70,000 people worldwide (WHO official records as of May 1, 2020). Currently, patients are treated with a combination of respiratory support, large spectrum antibiotics, and eventually extracorporeal oxygenation [2]; however, the outcomes are unsatisfactory. On March 18, 2020, the first clinical trial of antiviral agents (lopinavir-ritonavir) was published revealing no benefits in hospitalized adults with severe COVID-19, as compared with standard care [3]. Thus, the search for therapeutic strategies that could reduce the severity and high mortality rates of COVID-19 is an important issue in public health.

One of the intriguing aspects of COVID-19 is that hypertension and diabetes are the most important risk factors for severe progression and mortality, exceeding by far chronic obstructive pulmonary disease and asthma, which could intuitively be regarded as the preexisting disorders with the highest risk for unfavorable COVID-19 progression [2, 4]. We asked whether the mechanisms related to the viral infection could explain the high association between severe progression of COVID-19 and hypertension and diabetes. We found that SARS-CoV, responsible for the outbreak of SARS during the early 2000s, uses the enzyme ACE2 as a receptor for human cell entry [5]; in addition, a study published on March 6, 2020, revealed that SARS-CoV-2 also binds to ACE2, using the same mechanism to enter cells [6].

ACE2 catalyzes the inactivation of angiotensin II (AII) [7], and its putative inhibition by SARS-CoV-2 could result in increased systemic levels of AII. Patients with both hypertension and diabetes have abnormalities in the renin-angiotensin system, and this could increase the risk of circulatory, renal, and electrolytic abnormalities that, hypothetically, could result in increased severity of COVID-19.

However, the detailed evaluation of clinical and laboratory records from patients with severe COVID-19 shows low rates of circulatory abnormalities (shock was present in only 13% of patients with severe evolution of the disease), kidney failure (6%), and electrolytic abnormalities (virtually absent). These findings strongly suggest that increased AII levels do not explain the severity of the illness [2].

Another important function of the ACE system is the inactivation of the potent inflammatory substance bradykinin [8]. Accumulation of bradykinin in the lungs is a common side effect of ACE inhibitors leading to cough [9]. In animal models, the inactivation of ACE2 leads to severe acute pneumonitis in response to lipopolysaccharide (LPS), and the inhibition of bradykinin almost completely restores the lung structure [10].

In COVID-19 patients, rapidly progressing pneumonia is the main clinical feature associated with severe outcomes [2]. This is accompanied by high levels of C-reactive protein, indicating the magnitude of the inflammatory response [2]. Thus, we hypothesized that increased bradykinin, and not AII, is the main link between SARS-CoV-2 inhibition of ACE2 and severity of the disease. The abnormalities of the ACE system in hypertensive and diabetic patients, potentially associated with the chronic use of ACE inhibitors, could predispose to a massive increase in lung bradykinin leading to rapidly progressing pneumonia.

Currently, inhibition of bradykinin is the main therapeutic approach to treat a rare disease known as hereditary angioedema [11]. There are three major pharmacological approaches used to dampen bradykinin activity: (i) inhibition of bradykinin synthesis using either the human-derived inhibitor of C1e/kallikrein [12] or (ii) a synthetic inhibitor of kallikrein [13] and (iii) inhibition of bradykinin receptor 2 [14]. We propose the ITHACA trial (COVID-19 Treated by c1 esterase inHibitor And iCAtibant) to assess the efficacy and safety of the inhibitor of C1e/kallikrein and the inhibitor of bradykinin receptor 2 compared to standard care alone in the treatment of patients with pneumonia caused by COVID-19. We have designed a three-arm study to increase efficiency and reduce costs, since the same control arm will be used for comparison with two different drugs. In addition, this design increases the likelihood that the participant will be allocated to a treatment arm, which usually increases the chance of enrollment.

### Objectives {7}

The objective is to evaluate the efficacy and safety of each C1 esterase inhibitor plus standard care and use of icatibant plus standard care vs. standard care alone in hospitalized adult patients with pneumonia caused by COVID-19.

### Trial design {8}

**Fig. 1:**
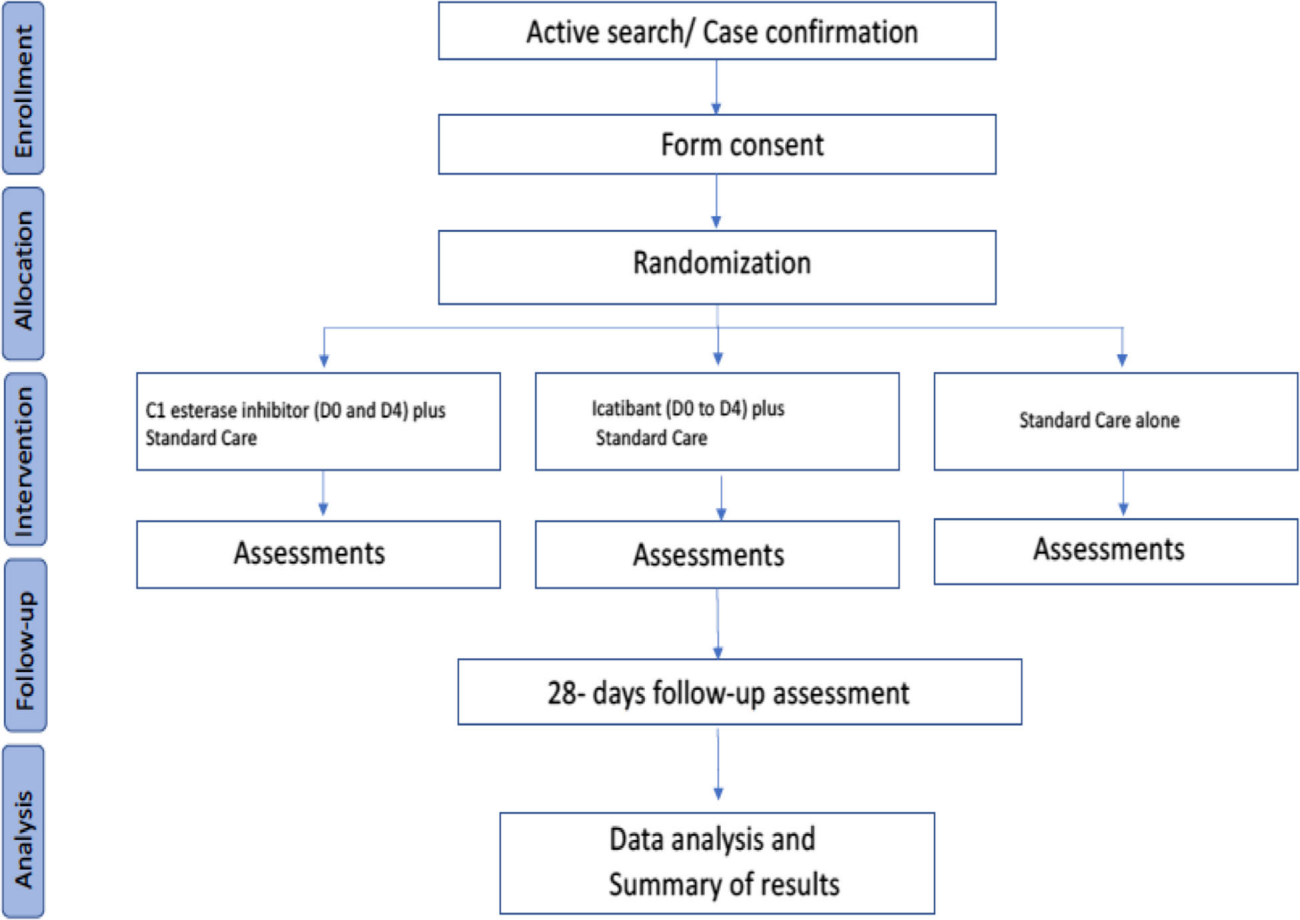
Graphic representation of the Ithaca Study design

This is a phase II/II, single-center, three-armed parallel-group, open-label, active control superiority, randomized clinical trial. We will use a 1:1:1 allocation ratio to C1 esterase inhibitor plus standard care vs. icatibant plus standard care vs. standard care alone. This study will follow the principles of the Declaration of Helsinki. The trial design is depicted in Fig. 1.

## Methods: participants, interventions, and outcomes

### Study setting {9}

This is a single-site study, and all procedures will be conducted at the Clinical Hospital at the University of Campinas in wards and intensive care units specifically focused on the care of patients with COVID-19. The expected enrollment start date is April 21, 2020, and the expected end date is July 21, 2020.

### Eligibility criteria {10}

Once all inclusion criteria have been met and the patient’s physician agrees to the patient’s participation in the study, a study staff member will apply the consent form and only then will the patient be included in one of the three study arms according to the randomization procedures.

The drugs under study, as well as the standard care treatment, will be prescribed by the attending physician of the hospital.

The following are the inclusion criteria:

1. Patients aged ≥ 18 years at the time of signing the consent form
2. ≤ 12 days since the onset of the symptoms until the start of the treatment
3. SARS-CoV-2 diagnosis by RT-PCR method
4. Pneumonia confirmed by computed tomography of the chest
5. Hospitalized patients with a SpO_2_ ≤ 94% in ambient air or PaO_2_/FiO_2_ ≤ 300 mmHg
6. The willingness of study participants to accept randomization to any assigned treatment arm
7. The patient or responsible (in the case of incapacitated adults) must have signed the consent form
8. The patient must agree not to enroll in any other experimental study prior to completing the 28-day follow-up

The following are the exclusion criteria:

1. Pregnancy or breastfeeding (pregnancy beta-HCG test will be performed in women of childbearing age).
2. Known severe renal impairment (estimated glomerular filtration rate ≤ 30 ml/min/1.73 m^2^), patients receiving continuous renal replacement therapy (hemodialysis or peritoneal dialysis), or previous renal transplant.
3. Known severe liver disease (AST or ALT 5× above the reference value).
4. Patients diagnosed with HIV infection or patient with any other immunodeficiencies.
5. Patients with a previous diagnosis of cancer.
6. Patients with a previous diagnosis of hereditary angioedema.
7. Patients with previous ischemic myocardial disease.
8. Patients with previous thromboembolic disease.
9. The assisting physician considers that the participation of the patient in the study is not appropriate, either because it is not of clinical interest or because of any condition that does not allow the protocol to be followed safely.
10. The patient will be transferred to any other hospital before the 28 days of follow-up or before discharge from the hospital.
11. Receipt of any experimental treatment for COVID-19 virus infection within 30 days prior to molecular screening.

### Who will take informed consent? {26a}

Study staff will obtain signed informed consent from the eligible patient or the responsible (in the case of incapacitated adults). The responsible will be the spouse/husband (or partner) unless there has been a legal or de facto separation, as determined by Art. 1.775 of the Brazilian Civil Code. In the absence of such persons, the burden will fall on the parents or descendants.

### Additional consent provisions for collection and use of participant data and biological specimens {26b}

The consent form contains provisions for the collection of research data and blood samples for the current study, but eventual ancillary studies, which could utilize the material stored in a biorepository, will be subject to the participant’s prior consent and approval of the new project by the local research ethics committee.

## Interventions

### Explanation for the choice of comparators {6b}

This study will have two active arms. The first one is C1 esterase inhibitor plus standard care. The second study arm is icatibant, a bradykinin receptor 2 inhibitor, plus standard care. Our hypothesis is that increased bradykinin is the main link between SARS-CoV-2 infection and inhibition of angiotensin 2-converting enzyme (ECA2), resulting in respiratory failure. Therefore, we chose the C1 esterase inhibitor and icatibant because they are the only two drugs that act in the inhibition of bradykinin that have been approved in Brazil. Both drugs are currently approved for use in humans for the treatment of hereditary angioedema, with good efficacy and a low side effect profile.

To date, there is no effective treatment for pneumonia caused by COVID-19 to include as an active control comparator. Thus, the third arm, which will be the comparator for the first two, will comprise standard care alone. The standard care will always involve the use of antibiotics intravenously and prophylactic antithrombotic therapy. Oxygen support, non-invasive and invasive mechanical ventilation, vasopressor use, and renal support therapy will also be provided as part of standard care, but will be used according to medical judgment.

Given that the COVID-19 pandemic is an emergency health problem worldwide, we chose not to use a placebo, since its formulation would delay the study schedule and increase costs.

### Intervention description {11a}

#### Group A (experimental) plus standard care

Firazyr® [Icatibant acetate 30 mg (with a concentration of 10 mg/mL—total 3 ml)] prefilled syringe delivers 30 mg of icatibant as icatibant acetate. Subcutaneous injection in the abdominal area was administered at intervals of 8 h for 4 days. This dose has been used successfully for the management of ACE inhibitor-induced angioedema and hereditary angioedema {Baş, 2015, A randomized trial of icatibant in ACE-inhibitor-induced angioedema}{Cicardi, 2010, Icatibant’, a new bradykinin-receptor antagonist’, in hereditary angioedema}. Drug-related adverse events occurred in 14–15% of the individuals receiving icatibant in the FAST-1 and FAST-2 trials, mainly injection site reaction, although with 11% of serious adverse events in FAST-2 trial, which were gastroenteritis and hypertensive crisis, laryngeal attack of angioedema requiring intubation, abdominal attack of angioedema, and cholelithiasis.

#### Group B (experimental) plus standard care

Berinert® [plasma-derived C1 esterase inhibitor (human)] will be administered at a dose of 20 IU/kg body weight on day 1 shortly after recruitment and on day 4. Each vial contains 500 IU of C1 esterase inhibitor as a lyophilized product for reconstitution with 10 mL of sterile water for injection, USP provided, following datasheet instructions. Berinert® is indicated for the treatment of acute hereditary angioedema (HAE) attacks at a dose of 20 IU/kg. The IMPACT-1 trial established the efficacy and security of Berinert® 20 IU/kg compared with placebo [15]. The most commonly observed adverse events reported on the Berinert® prescribing information are gastrointestinal symptoms [16]. Among 125 subjects enrolled in the IMPACT-1 trial, the treatment-related severe adverse reactions that occurred in 5 individuals treated with 20 IU/kg body weight were laryngeal edema, facial attack, swelling, exacerbation of hereditary angioedema, and laryngospasm.

#### Group C (active control)

The standard care will always provide antibiotics intravenously and prophylactic antithrombotic therapy. Oxygen support, non-invasive and invasive mechanical ventilation, vasopressor use, and renal support therapy will also be provided as part of standard care, but only according to medical judgment.

### Criteria for discontinuing or modifying allocated interventions {11b}

1. Withdrawal of consent. This item follows the Declaration of Helsinki, in which the patient has the right to withdraw his consent without any prejudice regarding the treatment offered to him/her by the clinical provider.
2. Grade 4 adverse reaction (life-threatening consequences) as stated by Common Terminology Criteria for Adverse Events (CTCAE) v6.0 used for oncology drugs by the National Cancer Institute (NCI).
3. Protocol violation.
4. Allergic reactions to any drug or any other situation deemed to jeopardize patient safety.

### Strategies to improve adherence to interventions {11c}

We consider that it is not necessary to draw up a plan of adherence to the interventions, since the patient will be hospitalized for the entire follow-up time and the healthcare professionals will administer the drugs (intravenous or subcutaneous) and standard care according to medical prescription.

### Relevant concomitant care permitted or prohibited during the trial {11d}

All usual intensive care management procedures are permitted and will be determined by the clinical providers, in accordance with the Asian Critical Care Clinical Trials Group [17], but the patient will not be allowed to be on another experimental protocol, e.g., chloroquine or others.

### Provisions for post-trial care {30}

The patients in our study will receive the most suitable standard care treatment, plus the proposed medicines, according to the allocation arm, and will be monitored by the medical team of the Clinics Hospital of the University of Campinas during the 28 days of follow-up (or hospital discharge), or as long as necessary. Therefore, patients will receive medical care during the trial follow-up itself in relation to serious adverse events (SAE). Regarding adverse events in general, we also believe that most of them should occur during this period; however, the post-trial care of the patient is ensured through free care at the Clinics Hospital of the University of Campinas, both via the emergency room and outpatient care, following the principle of universality of the Brazilian Health System (SUS).

Regarding the results on efficacy, if there is a positive impact of any of the medications investigated on hospitalized patients, the benefit on the participants of the study will be assessed during the follow-up period. During the trial, we intend to maintain a dialog with competent health authorities (at municipal, state, and federal levels) in order to enable the negotiation of more affordable prices for medicines, as well as coordinate their acquisition and distribution to the target patients.

### Outcomes {12}

#### Primary outcome

1. Time to clinical improvement (TTCI)—the TTCI was defined as proposed by the Cap-China Network [18] and thereafter utilized in the LOTUS China trial [3] and recommended by the World Health Organization (WHO) [19]. Briefly, TTCI refers to the time from randomization to an improvement of two points on the seven-category ordinal scale stated below [3] or live discharge from the hospital:
  - Not hospitalized with the resumption of normal activities
  - Not hospitalized, but unable to resume normal activities
  - Hospitalized, not requiring supplemental oxygen
  - Hospitalized, requiring supplemental oxygen
  - Hospitalized, requiring nasal high-flow oxygen therapy and non-invasive mechanical ventilation
  - Hospitalized, requiring ECMO, invasive mechanical ventilation, or both
  - Death

#### Secondary outcomes

1. Percentage of patients on a seven-category scale on days 7, 14, and 21
2. Duration (days) of mechanical ventilation
3. Duration of hospitalization (days) of survivors
4. Need for oxygen support (days)
5. Time from randomization to hospital discharge (days)
6. Time from randomization to death (days)
7. Occurrence of acute kidney injury, defined as an increase in creatinine 1.5 times above the baseline
8. Frequency of severe adverse events as defined by the National Cancer Institute Common Terminology Criteria for Adverse Events (CTCAE), version 5.0

### Participant timeline {13}

The participant timeline is depicted in Table 1.

**Table 1:** Timeline of events in the trial

	Enrollment	Allocation	Post-allocation				Discharge	Close-out
Time point	− 1	0	D1–D4	D5–D7	D8–D14	D15–D28		
Enrollment								
Screening	x							
Eligibility criteria	x							
Informed consent	x							
Allocation		x						
Randomization		x						
Baseline data collection		x						
Interventions								
Icatibant/std care			x					
C1 inhib./std care			x					
Std care	x	x	x	x	x	x	x	
Outcome/safety assessments								
CT of the chest	x				X (D14)	X (D28)	X	
Primary outcome			x	x	x	x	x	
Clinical symptoms		x	x	x	x	x	x	
Vital signs	x	x	x	x	x	x	x	x
ECG				x				
Adverse events	x	x	x	x	x	x	x	x
Time mechanical ventilation								x
Death								x
Length of stay								x
Laboratory tests*	Only every 4 days or at the discretion of the assistant physician							
RT-PCR SARS-CoV-2	x							
Bradykinin		x	x				x	
SOFA score		x	x	x	x	x	x	
Blood routine		x	x	x	x	x	x	
Coagulation routine		x	x	x	x	x	x	
CRP		x	x	x	x	x	x	
Liver function		x	x	x	x	x	x	
Kidney function		x	x	x	x	x	x	
d-dimer		x	x	x	x	x	x	
High-sensitivity troponin		x	x	x	x	x	x	
Pro-BNP		x	x	x	x	x	x	
Aspergillus antigen		x	x	x	x	x		

### Sample size {14}

**Fig. 2:**
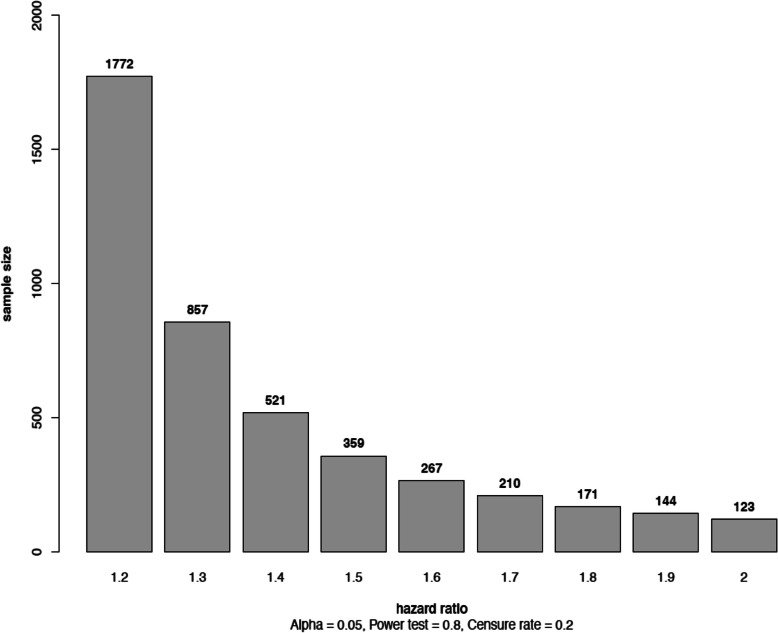
Graphic representation of the sample size definition

We used information from the previously published LOTUS [3] trial evaluating the effect of lopinavir-ritonavir on time to clinical improvement (TTCI) [censored at day 28], since there is no information on the effect of icatibant or C1 esterase inhibitor for this primary outcome. This approach resulted in a hazard ratio of 1.24 for clinical improvement. It is also relevant to take into account the emergency situation and the high costs involved. The sample size was determined based on the formula for the proportional hazard regression model (Schoenfeld, 1983). We assumed a 5% type 1 error (alpha), power test of 80%, drop-out (censure) rate of 20%, and hazard ratio between treatment and the standard care alone varying from 1.2 to 2.0. To extend the calculations for a three-arm trial, we considered the three groups of the same size and the comparison of the two treatments separately to standard care alone, but without comparing the two treatments to each other. In other words, the same standard care alone group will be compared with each treatment group. Additionally, we set alpha at 2.5% to allow for multiple testing and multiplied the sample size by 1.5. Figure 2 presents the total sample size required for this setup and according to different hazard ratios. Assuming a hazard ratio of 1.9, we obtained a final sample size of 174 (58 per group).

### Recruitment {15}

A physician will recruit patients via an active search. She/he will perform visits three times a day (9 am, 3 pm, 9 pm) to the sites of the hospital that will receive patients with suspected COVID-19, such as the emergency room, COVID-19-dedicated wards, and ICUs. She/he will also determine whether the patient meets the eligibility criteria for the study, expedite the exams required to comply with the inclusion criteria (RT-PCR for SARS-CoV-2 and computed tomography of the chest), and will make immediate contact with the study coordinator for enrollment.

## Assignment of interventions: allocation

### Sequence generation {16a}

We will use a software-based approach (www.sealedenvelope.com) to generate the allocation sequence using a blocked randomization with randomly selected block sizes, with 3, 6, or 9 subjects per block. To reduce the probability of imbalance between the groups with regard to severity, we have set up a stratification approach using two strata: (1) no oxygen support needed or oxygen support < 3 L/min delivered by nasal catheter; (2) high-flow oxygen support delivered by nasal catheter between 3 L and 6 L/min or up to 10 L/min by non-rebreather face mask, 35% oxygen through a Venturi mask, non-invasive ventilation, or mechanical ventilation or eventually ECMO.

### Concealment mechanism {16b}

Sealed and sequentially numbered opaque envelopes, containing the allocation arm, will be prepared in advance according to the randomization list. These envelopes will be stored in one of the ICUs dedicated to COVID-19 at the Clinics Hospital of the University of Campinas. Once the eligibility criteria have been met and the form has been signed, the study coordinator (using a central telephone or message) will double-check with the assistant physician the exact envelope number corresponding to the patient’s allocation following an established standard procedure. The same physician should then open the envelope and soon after prescribe the therapy according to the arm specified in the envelope.

### Implementation {16c}

The randomization list will be generated by an independent researcher, who will not have any other involvement in the study. The project coordinator and investigators in each unit of the hospital (wards, emergency department, and ICUs) will enroll the participants after contacting (central telephone, message, in-person) the physician responsible for the active search in the hospital reporting eligible patients. An attending physician will allocate the patient by opening the sequential envelope in virtual contact with the central telephone and assign participants to interventions.

## Assignment of interventions: blinding

### Who will be blinded {17a}

This is an open-label trial because neither the trial participant nor the care providers will be blinded. However, the data entry staff and data manager will be blinded. The data will be sent to the data entry staff by code, and the study arm will not be specified. The data entry staff and data managers are not part of the hospital staff and will not have access to any clinical data belonging to the patients. The Data Safety Monitoring Board (DSMC) will be unblinded.

### Procedure for unblinding if needed {17b}

This does not apply because it is an open-label study. We consider that there will be no need to unblind data entry staff or data managers, as they are not involved in the course of patient care.

## Data collection and management

### Plans for assessment and collection of outcomes {18a}

Baseline data, other trial data, and outcome data will be collected by all investigators participating in this study and will be stored in the Research Electronic Data Capture (REDCap) platform. The investigators will be trained to apply the seven-category ordinal scale in order to collect the outcome data. Data will be collected daily by one investigator on site using a datasheet available online and in print form.

### Plans to promote participant retention and complete follow-up {18b}

Volunteers can withdraw their consent during any stage of the study, without prejudice to their individualized treatment. In this situation, the investigator will clarify any doubts and inform them of the potential benefits of being in the study. The patient’s withdrawal will be reported in the eCRF, and the outcome will be assessed on the day of withdrawal and the patient will be invited to return if possible for an additional visit after the study has closed, in order to collect data on adverse events. Patients excluded due to adverse events will be followed up to day 28, or until necessary. Patients with any deviation in the intervention, due to risk or request, will have their outcomes collected normally according to the proposed timeline.

### Data management {19}

Each subject allocated in the study will be given a unique code generated by the statistician responsible for the randomization list (all possible identifiers will be removed). This code will be maintained by the PI and the lead coordinator in a password-protected file, which will not be accessible to the data management team or other investigators. All data included (outcome, questionnaires, clinical data, computed tomography, biochemical analysis) will be retrieved from the online official medical record of the hospital and will be uploaded into REDCap. Data will be collected daily by one investigator on site using a datasheet available online and in print form. Collected data will be sent by email to two researchers in the data management team in order to establish double data entry, as well as to an independent company reviewing the quality of the data, and the data management team, which is responsible for range checks for data values. A Standardized Operational Protocol for data collection will be available as supplementary material.

### Confidentiality {27}

The personal information of the allocated patient and the enrolled patient collected by the investigator will be identified by the same individualized code generated for the randomization list. The data will be entered into the CRF using this code. The identification of this code will not be known by the researchers and will be kept in a password-protected document in a spreadsheet maintained in a cloud accessible by the PI and the coordinator.

### Plans for collection, laboratory evaluation, and storage of biological specimens for genetic or molecular analysis in this trial/future use {33}

The nasopharyngeal/oropharyngeal swabs for RT-PCR will be collected by the patient care provider team and sent to the hospital’s laboratory in accordance with the local protocol, as well as for routine exams. RNA from whole blood, serum, and plasma samples will be stored for future use in ancillary studies, which will need approval from local ethics committees.

## Statistical methods

### Statistical methods for primary and secondary outcomes {20a}

All our statistical analyses will follow the intention-to-treat principle. For the primary outcome, we will use the Kaplan-Meier estimator and log-rank test to respectively estimate and compare the survival curves, followed by a Cox proportional hazards model to adjust for baseline explanatory variables in the final data analysis. Multiple imputation will be used to impute missing data. The following covariates will be settled: age, sex, duration of symptoms before enrollment, use of oxygen support prior to enrollment, and presence of relevant comorbidities. This analysis will be performed to compare each treatment arm, that is, the icatibant plus standard care group and the C1 esterase group inhibitor plus standard care, with the standard care group alone. In the analysis of secondary endpoints involving time to event, such as duration of mechanical ventilation (days) and length of hospitalization in survivors (days), we will also use the Cox proportional hazards model. The logistic regression model will be used to analyze secondary binary endpoints.

The statistical analysis plan (SAP) containing all the details of the analysis will be finalized and made available to the DSMC before the final analysis.

### Interim analyses {21b}

We will perform an interim analysis for effectiveness, futility, and safety when we have reached 50% of the events between all groups (three arms).

The Cox proportional hazards model will be used to evaluate the primary outcome in this interim analysis. The logistic regression model will be used to evaluate the occurrence of severe adverse events as a secondary endpoint.

An independent statistician will perform the interim analyses, and the results will be evaluated by the DSMC.

### Methods for additional analyses (e.g., subgroup analyses) {21b}

Additional subgroup analyses will be performed for gender (male/female), age group (individuals up to 60 years old/individuals 60 years old or older), and severity of the disease at the randomization time (less severe presentation: patient in ambient air or using O_2_ in catheter; more severe presentation: use of O_2_ high flow, NIV, MV, or ECMO).

The analysis of all these subgroups for the primary endpoint will be done using the Cox proportional hazards model. A mortality analysis will also be performed on each of these subgroups using the logistic regression model. The analysis of all the subgroups mentioned here will be performed for the comparison of both icatibant plus standard care vs. standard care alone and for the comparison of C1 esterase Inhibitor plus standard care vs. standard care alone.

### Methods of analysis to handle protocol non-adherence and any statistical methods to handle missing data {21c}

We will use multiple imputation for missing data. A per-protocol analysis will be used to check the robustness of the results obtained primarily by the intention-to-treat principle.

### Plans to give access to the full protocol, participant-level data, and statistical code {31c}

After the study has ended, we intend to make available the full protocol and the statistical code through scientific publication.

## Oversight and monitoring

### Composition of the coordinating center and trial steering committee {5d}

#### Coordinating center

Clinics Hospital, University of Campinas, Campinas, São Paulo, Brazil

#### Trial steering committee

- Maria Luiza Moretti, Department of Internal Medicine, School of Medical Sciences, University of Campinas, Campinas, São Paulo, Brazil
- Lício Augusto Velloso, Obesity and Comorbidities Research Center, Department of Internal Medicine, School of Medical Sciences, University of Campinas, Campinas, São Paulo, Brazil
- José Carlos de Lima-Júnior, Obesity and Comorbidities Research Center, Department of Internal Medicine, School of Medical Sciences, University of Campinas, Campinas, São Paulo, Brazil
- Flávia Fagundes Bueno, Obesity and Comorbidities Research Center, Department of Internal Medicine, School of Medical Sciences, University of Campinas, Campinas, São Paulo, Brazil
- Tatiana Benaglia, Department of Statistics, Institute of Mathematics, Statistics, and Scientific Computation, University of Campinas, Campinas, São Paulo, Brazil

#### Trial operation committee

- Elie Mansour, Department of Internal Medicine, University of Campinas, Campinas, São Paulo, Brazil
- Antonio L. E. Falcao, Department of Surgery, University of Campinas, Campinas, São Paulo, Brazil
- Thiago Martins Santos, Department of Internal Medicine, University of Campinas, Campinas, São Paulo, Brazil

#### Data management team

- Clarity Health Intelligence Co., Ltd.
- Milena Monfort-Pires, Obesity and Comorbidities Research Center, University of Campinas, Campinas, São Paulo, Brazil
- Bruna Bombassaro, Obesity and Comorbidities Research Center, University of Campinas, Campinas, São Paulo, Brazil
- Eliana Pereira de Araújo, School of Nursing, University of Campinas, Campinas, São Paulo, Brazil

#### Trial monitoring

Clarity Health Intelligence Co., Ltd.

#### Clinical research organization

Clarity Health Intelligence Co., Ltd.

### Composition of the data monitoring committee, its role, and reporting structure {21a}

Given that this study is of high interest to the scientific community within the context of the pandemic and that it may deeply affect the clinical practice, we have chosen to adopt a Data Safety Monitoring Committee (DSMC). In addition, there are concerns regarding the use of experimental drugs in the specific condition studied. The DSMC will aim to assess the continuing validity of the trial (critical efficacy endpoints), monitor adverse events, ensure clinical equipoise, and monitor protocol compliance, data quality, and participant recruitment. The DSMC will be composed of clinicians, the study sponsor, and at least one biostatistician. The agenda of group meetings and the template to be followed will be defined by the team itself. This committee will have an advisory role and is composed of Daniel Munhoz and Luiz Sergio F. Carvalho.

### Adverse event reporting and harms {22}

The project investigators will prepare reports according to the adverse event data collected by themselves.

Both adverse events (AEs) and serious adverse events (SAEs) will be collected throughout the follow-up period, from the time of informed consent to day 28 of evaluation. The serious adverse events that occur after day 28 will be evaluated by the investigators and the DSMC to determine whether they have any relation with the experimental drug under study.

All SAEs will be accompanied by study physicians until there is total or at least significant improvement.

Following the International Conference on Harmonization Guidelines for Good Clinical Practice, all AEs will be investigated in detail and included in an electronic CRF (REDcap). The information collected will include the following: start date of possible AE, reported signs and symptoms, relationship between the reported event and the drugs studied, outcome of the adverse event, and the dates on which the data were evaluated by the DSMC.

### Frequency and plans for auditing trial conduct {23}

An audit of this trial will be carried out by an independent group with the aim of identifying problems more promptly in any of the phases of this protocol and thus correct them as soon as possible, in accordance with the principles of the International Conference on Harmonization Guidelines for Good Clinical Practice.

This group should make an evaluation before starting the trial to verify that the study coordinators are able to carry out the inclusion and the management of the data appropriately. During the trial, they should also check whether the entire study protocol is occurring appropriately, including recruitment, enrollment, and allocation of participants; adherence of hospital staff to interventions and health care procedures; reporting of harms; and data collection and management. There should be one visit of the monitoring group to the Clinics Hospital of the University of Campinas during the study to check whether such criteria are being fulfilled and also with an educational function in order to encourage all those involved in the research to adhere to the protocol.

More detailed information about the audit process of this trial can be found in the Manual Audit.

### Plans for communicating important protocol amendments to relevant parties (e.g., trial participants, ethics committees) {25}

Any change in the study protocol involving the studied population, study design, eligibility criteria, patient allocation, randomization process, outcomes, sample size, statistical data analysis, procedures of study, or administration of study data should generate protocol amendments, which will always be submitted for analysis by the Ethics Committee/institutional review board (IRB). The changes will only be implemented after approval by this committee, unless this change may harm the patient’s safety.

All protocol amendments will also be sent to the DSMC.

### Dissemination plans {31a}

The study results will be published in conferences, a pre-print database, and peer-reviewed journal publications.

We also hope that the data will be disseminated to the health authorities and the lay audience, so that we can have wide public knowledge of these results. We will prepare a one-page report, which will be informative and visual, to be delivered to the patients who participated in the study.

## Discussion

So far, there are no specific treatments for COVID-19. There is great concern regarding the duration of hospital treatment required in severe forms, because in the context of a pandemic, prolonged hospitalization means that health services may not be able to meet the demand generated by the disease. Therefore, we consider that one of the main aims for the treatment of severe pneumonia caused by COVID-19 should be to reduce the length of stay. Recent studies have shown that pulmonary inflammation and extensive lung damage in SARS patients can lead to more severe conditions [1, 2]. Furthermore, researchers have shown that SARS-CoV-2 binds to the angiotensin 2-converting enzyme (ECA2) to gain access to cells. This enzyme is fundamental both in the inactivation of angiotensin II (ANGII) and in the inactivation of bradykinin. The accumulation of bradykinin in the lungs is a common effect after the use of ECA2 inhibitors. In animal models, ECA2 inactivation leads to severe pneumonitis, and bradykinin inhibition fully recovers lung function and structure [9].

Several previous clinical trials have already shown the efficacy of both C1 esterase and icatibant, both bradykinin inhibitors, for use in a condition called hereditary angioedema. Considering our hypothesis that the release of bradykinin could be responsible for the hyperinflammatory state of the airway in SARS-CoV-2 infection and that its inhibition could reduce such inflammation, we imagine that the use of such drugs would help reduce the complications caused by COVID-19 pneumonia and consequently reduce the duration of hospitalization. This would mean a great gain for the health system since it would allow greater patient turnover, besides reducing the chance of secondary infections inherent in a long hospitalization.

We chose to define our study population as patients hospitalized due to COVID-19 pneumonia precisely because we considered that patients with severe disease would be those who would benefit most from reduced hyperinflammatory status. The choice of outcome as the time to event (improvement of the ordinal scale of seven items or hospital discharge) was based on previous clinical studies (LOTUS and Remdesivir trial). We consider that this outcome will allow us to evaluate whether in fact the drugs tested contribute to a reduction of morbidity over time. In addition, it will help us to infer whether the use of intervention could mean a shorter stay. As the outcome is the same as in previous studies, we will also have the benefit of literature comparison.

We will be using a multi-arm design to increase study effectiveness and reduce costs. C1 esterase and icatibant were selected because they are the only two drugs that inhibit the bradykinin pathway that have been approved for use in patients in Brazil. In addition, they require short-term use (only 4 days) and already have a safe and effective dose established for use in hereditary angioedema.

We chose standard care as the comparator only because we believe that there are no clinical trials with sufficient impact to indicate any specific treatment for COVID-19 pneumonia. Therefore, the use of any specific drug in our study would be unethical. The use of a placebo is not possible, since the development of two placebos identical to the interventions, one of intravenous infusion and another of subcutaneous infusion, would require a lot of time and would significantly increase costs. Blinding is also not possible in this study. We cannot blind the assisting physician either because of safety issues or because she/he will be responsible for prescribing the experimental drug. In addition, the absence of a placebo would make it much easier for the patient to guess that she/he was selected for the intervention. This is the first attempt at evaluating the use of bradykinin pathway inhibitors in pneumonia caused by COVID-19. We believe that the results will be of great importance for hospital management of the disease at the global level.

## Trial status

Version 2.0, November 17, 2020, recruiting; the expected end date is April 2021.

## Data Availability

The final data of the study and the generated database will be available for access by all investigators and the entire scientific community.

## Abbreviations

ACE2: Angiotensin 1-converting enzyme 2
ACE: Angiotensin-converting enzyme
AE: Adverse event
ALT: Alanine aminotransferase
ANG II: Angiotensin II
AST: Aspartate aminotransferase
Beta-HCG: Beta human chorionic gonadotropin
C1: Complement 1
COVID-19: Coronavirus disease 2019
CRF: Case report form/clinical research flowchart
CRP: C-reactive protein
CT: Computed tomography
CTCAE: Common Terminology Criteria for Adverse Events
DSMC: Data Safety Monitoring Committee
ECG: Electrocardiogram
ECMO: Extracorporeal membrane oxygenation
eCRF: Electronic case report form
FAPESP: Sao Paulo Research Foundation
FiO_2_: Fraction of inspired oxygen
HIV: Human immunodeficiency virus
ICU: Intensive care unit
IRB: Institutional review board
ITHACA: COVID-19 Treated by c1 esterase inHibitor And iCAtibant
IU: International unit
LOTUS: A trial of LOpinavir-RiTonavir in adUlts hospitalized with Severe COVID-19
LPS: Lipopolysaccharide
MV: Mechanical ventilation
NIV: Non-invasive ventilation
PaO_2_: Partial pressure of oxygen
PI: Principle investigator
ReBec: Brazilian Clinical Trials Registry
REDCap: Research Eletronic Data Capture
RCT: Randomized clinical trial
RNA: Ribonucleic acid
RT-PCR: Reverse transcription PCR
SAE: Serious adverse event
SAP: Statistical analysis plan
SARS-CoV: Severe acute respiratory syndrome coronavirus
SOFA: Sequential organ failure assessment
SOPs: Standard operation procedures
SpO_2_: Partial saturation of oxygen/pulse oximeter oxygen saturation
SUS: Sistema Único de Saúde (to refer to Brazilian Health System)/integrated health system
TTCI: Time to clinical improvement
USA: United States of America
USP: United States Pharmacopeia
UTN: Universal trial number
WHO: World Health Organization
